# Deciphering Hydrodynamic and Drug-Resistant Behaviors of Metastatic EMT Breast Cancer Cells Moving in a Constricted Microcapillary

**DOI:** 10.3390/jcm8081194

**Published:** 2019-08-09

**Authors:** Binita Nath, Anil P. Bidkar, Vikash Kumar, Amaresh Dalal, Mohit Kumar Jolly, Siddhartha S. Ghosh, Gautam Biswas

**Affiliations:** 1 Department of Mechanical Engineering, Indian Institute of Technology Guwahati, Guwahati 781 039, India; 2 Department of Biosciences and Bioengineering, Indian Institute of Technology Guwahati, Guwahati 781 039, India; 3 Centre for BioSystems Science and Engineering, Indian Institute of Science Bangalore, Bangalore 560 012, India; 4 Department of Mechanical Engineering, Indian Institute of Technology Kanpur, Kanpur 208 016, India

**Keywords:** metastasis, constricted microchannel, hydrodynamic parameters, breast cancer cells, epithelial to mesenchymal transition, EMT, mesenchymal to epithelial transition, MET, cell viability

## Abstract

Epithelial to mesenchymal transition (EMT) induces cell migration, invasion, and drug resistance, and consequently, contributes to cancer metastasis and disease aggressiveness. This study attempted to address crucial biological parameters to correlate EMT and drug-treated cancer cells traversing through microcapillaries, reminiscent of metastatic conditions. MDA-MB-468 breast cancer cells induced to undergo EMT by treatment with 20 ng/mL of epidermal growth factor (EGF) were initially passed through several blockages and then through a constricted microchannel, mimicking the flow of invasive metastatic cells through constricted blood microcapillaries. EMT cells acquired enhanced migratory properties and retained 50% viability, even after migration through wells 10–15 μm in size and a constricted passage of 7 μm and 150 μm in length at a constant flow rate of 50 μL/h. The hydrodynamic properties revealed cellular deformation with a deformation index, average transit velocity, and entry time of 2.45, 12.3 mm/s, and 31,000 μs, respectively for a cell of average diameter 19 μm passing through one of the 7 μm constricted sections. Interestingly, cells collected at the channel outlet regained epithelial character, undergoing reverse transition (mesenchymal to epithelial transition, MET) in the absence of EGF. Remarkably, real-time polymerase chain reaction (PCR) analysis confirmed increases of 2- and 2.7-fold in the vimentin and fibronectin expression in EMT cells, respectively; however, their expression reduced to basal level in the MET cells. A scratch assay revealed the pronounced migratory nature of EMT cells compared with MET cells. Furthermore, the number of colonies formed from EMT cells and paclitaxel-treated EMT cells after passing through a constriction were found to be 95 ± 10 and 79 ± 4, respectively, confirming that the EMT cells were more drug resistant with a concomitant two-fold higher expression of the multi-drug resistance (MDR1) gene. Our results highlight the hydrodynamic and drug-evading properties of cells that have undergone an EMT, when passed through a constricted microcapillary that mimics their journey in blood circulation.

## 1. Introduction

Epithelial to mesenchymal transition (EMT) is a physiological phenotypic shift of epithelial to mesenchymal cells, where the breakdown of cell–cell and cell–extracellular matrix connections permits the migration of cells to distant locations [1]. The role of EMT is well documented in normal embryonic development, tissue regeneration, organ fibrosis, and wound healing [2]. Furthermore, EMT is involved in tumor progression with metastatic expansion and the generation of tumor cells with stem cell properties that play a major role in resistance to cancer treatment. Although mesenchymal cells possess increased migratory capacity, invasiveness, and greater resistance to apoptosis [3], the dynamics of EMT during invasion are yet to be fully elucidated to resolve the mystery of cancer metastasis. On the other hand, the reverse transition, i.e., mesenchymal to epithelial transition (MET), is attributed to the migrating mesenchymal cells once they reach their destination [4]. MET is thought to be crucial for the colonization of a metastatic niche by disseminated tumor cells. EMT and MET are not binary phenomena, and tumor cells can be in multiple hybrid states and express both epithelial and mesenchymal genes [5,6]. Such hybrid cells can move collectively as clusters and may be stem-like and metastatic compared with cells with a complete EMT phenotype [7,8]. Experimentally, EMT can be induced by adding growth factors such as epidermal growth factor (EGF), transforming growth factor beta (TGF-β), and hepatocyte growth factor (HGF); EGF and TGF-β induce EMT via Smad2/3 and ERK1/2 pathways [9,10]. Various processes are involved in initiating an EMT, including activation of transcription factor, expression of specific cell-surface proteins, re-formation and expression of cytoskeletal proteins, creation of extracellular matrix (ECM)-degrading enzymes, and changes in the expression of specific microRNAs [11]. The epithelial cells that undergo EMT and invade the bloodstream from the primary site often display alterations in gene expression and lose some epithelial characteristics, such as apical–basal polarity [12]. Important characteristics of EMT include downregulation of epithelial markers including E-cadherin, occludin, and claudin. Contrarily, increases in the levels of mesenchymal markers such as N-cadherin, vimentin, and fibronectin have been associated with EMT. The expression pattern of these genes can be tracked to study the behavior of the cells while transitioning from epithelial to mesenchymal or vice versa.

In most previous studies, the morphology and characteristics of cells undergoing EMT and MET have been studied under static conditions [13,14]. Hence, the physiological conditions in which the EMT cells traverse through the blood vessels or microcapillaries and undergo reverse transition at the secondary site are a very important area of investigation. An attempt to find the percentage viability and metastatic potency of the EMT cells after transiting through microcapillaries would reveal important information that could address many of the questions related to the complex phenomenon of cancer metastasis. Moreover, a detailed investigation of the treatment of cancer cells with drugs at different stages of their flow from the primary site to a secondary site undergoing EMT to MET transitions (through the capillaries) would be crucial for designing future theranostic devices aiming at curative or palliative treatment. Hence the dynamics of the motion, deformation, and behavioral changes of EMT cancer cells passing through microcapillaries still seem to be underexplored and require further attention.

**Figure 1:**
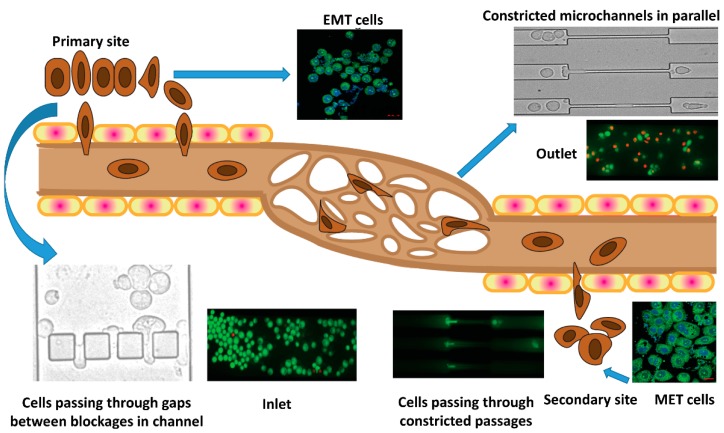
Schematic representation of the motion of metastatic cancer cells from the primary site to a distant secondary site through microcapillaries.

In this work, we attempted to address some of the questions related to the motion of EMT cancer cells through microcapillaries. For this purpose, a 2.85 mm long microchannel was fabricated using polydimethylsiloxane (PDMS), with an overall width of 184 μm. At a distance of 700 μm from the inlet, four 30 μm square blockages were inserted, varying the gap between the blockages in the range 10–15 μm. This complex configuration of the blocks and gaps helped us to mimic the broken basement membrane via which cancer cells invade the blood capillaries. Further, at a distance of 1000 μm from the blockages, a network of constricted channels each of width 7 μm, which mimic the blood microcapillaries, were introduced. The motion of the EMT cells through this complex network allowed the investigation of some hydrodynamic parameters along with some crucial biological assays. The deformation index, entry time, and transit velocity of the cells at different stages provide an understanding of the behavior of cells in microcapillaries. The EMT cells at the inlet, and the cells that were collected from the outlet and regrown (MET), were examined by analyzing several protein expressions, real-time polymerase chain reaction (PCR) analysis, and flow cytometric analysis. The viability of the cells was calculated using dye staining assays. A comparison between the migrating ability of EMT cells and epithelial cells was made by performing a scratch assay. Moreover, the metastatic potency of the EMT cells passing through the constricted channel was observed by performing colony formation assays. Whereas paclitaxel treatment resulted in decreased viability (IC_50_ = 98 nM) and colony formation ability of epithelial cells, paclitaxel-treated EMT cells showed a lower response to drug treatment. Furthermore, a thorough investigation to identify the effectiveness of drug treatment during various stages of cancer cell flow through microcapillaries was undertaken, which may prove very beneficial for scientists, oncologists, and cancer therapeutics. Figure 1 shows a graphical representation of the objectives of the present study.

## 2. Experimental Section

### 2.1. Cell Culture

Breast cancer (MDA-MB-468) cells were obtained from National Centre for Cell Science, Pune, India. These cells were maintained in a CO_2_ incubator (5% CO_2_) with humidified air. Cells were cultured in DMEM (Dulbecco’s modified Eagle’s medium) containing 10% FBS (fetal bovine serum) and antibiotic solution (penicillin and streptomycin). MTT (3-(4,5-dimethylthiazol-2-yl)-2,5-diphenyltetrazolium bromide), hEGF (human epidermal growth factor), and a GenElute Mammalian Total RNA Miniprep Kit were purchased from Sigma Aldrich. A cDNA synthesis kit and the cyanine dye SYBR Green were purchased from BioRad Laboratories. Alexafluor 488-tagged anti-vimentin antibody was obtained from Abcam.

### 2.2. EMT Induction in MDA-MB-468 Cells

Nearly confluent MDA-MB-468 cells were washed with phosphate-buffered saline (PBS), trypsinized, and counted in a Countess cell counter (Thermo Fisher Scientific). Cells were seeded in a six-well plate at a density of 2 × 10^5^ cells per well in 2 mL of medium (10% FBS). The six-well plates were incubated in a CO_2_ incubator for 24 h for complete attachment of the MDA-MB-468 cells. After 24 h, the medium in each well was replaced with a serum-free medium and the cells were again incubated for 12 h. Subsequently, the cells were treated with EGF at 10, 20, and 40 ng/mL in a serum-free medium. The morphology of the untreated and EGF-treated cells was monitored and images were captured in a microscope (Nikon Eclipse Ti-U). These EGF treated cells (MDA-MB-468 cells) are henceforth referred to as EMT cells.

### 2.3. Fabrication of Microchannels

**Figure 2:**
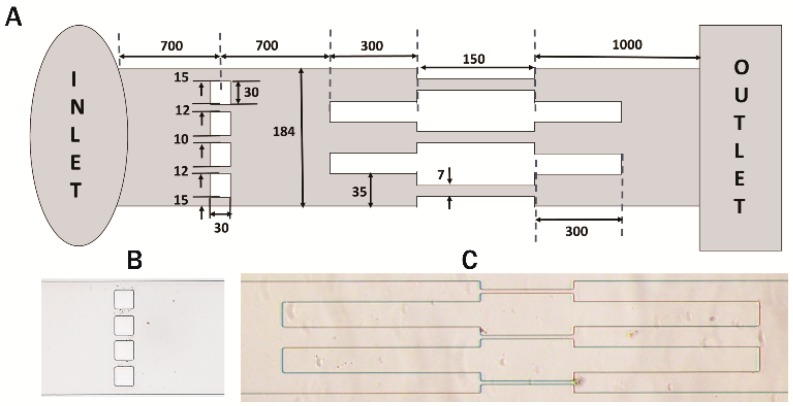
Design of the microchannel. (**A**) Schematic representation of the microchannel (not to scale); (**B**) magnified view of the 30 μm blockages; (**C**) magnified view of the series of constricted channels. All dimensions in μm.

An Su8 master silicon wafer was prepared in the CeNSE Department of IISc Bangalore, India, having imprints for 12 channels in the single master. All the channels were fabricated based on the design shown in Figure 2A. PDMS solution was prepared by mixing SYLGARD 184 silicone elastomer with a cross-linker in a ratio of 10:1. A nylon ring of inner diameter equal to the width of the Su8 master was used to make the mold. PDMS solution was poured over the Su8 master bounded by the nylon ring. Upon solidification of the PDMS layer, it was gently peeled off and individual channels were cut out using a surgical blade. The inlet and outlet were punched out using a punching tool. The open channels were then sealed by placing them over a glass slide after treatment with oxygen plasma.

### 2.4. Experimental Setup

Semi-rigid polyethylene tubing of outer diameter 1.09 mm and inner diameter 0.38 mm (Prolab Marketing, New Delhi, India) was connected to the inlet and outlet of the microchannel. The cells suspended in DMEM were filled into a syringe from an Eppendorf tube and the syringe was fitted in a syringe pump. The microscope and pump were connected to a power source. With activation of the syringe pump, the cells and the suspending medium started to fill the connecting tube and flow through the microchannel. In general, the average velocity of blood (usually measured in cm/s) varies from 0.03 to 40 cm/s as the blood flows through the vena cava, capillaries, and aorta [15]. The cells suspended in the medium were allowed to flow pass at a constant flow rate of 50 μL/h. The motion of the cells were observed and recorded at a high frame rate of 30,000–50,000 fps using the video module of Phantom PCC 2.8 software, manufactured by Vision Research (Wayne, NJ, USA). The videos were then deconvoluted to obtain images at required time instants. The supplementary videos are shown at a reduced speed of 200 fps for clarity.

### 2.5. Flow Cytometry for Vimentin Expression

MDA-MB-468 cells were seeded in a six-well plate at a density of 2 × 10^5^ cells per well. After EMT induction, the cells were washed with PBS, trypsinized, and collected in 1.5 mL tubes. The cells were fixed with 1 mL of formaldehyde (4% in PBS) at room temperature for 15 min. Thereafter, the cells were centrifuged and washed with PBS. Subsequently, permeabilization of the cell membranes was achieved by adding chilled methanol (90%) on ice. After 30 min of incubation on ice, the cells were centrifuged and washed with PBS. Alexafluor 488-tagged anti-vimentin antibody was added to the cells in 4% bovine serum albumin (BSA) solution, and then the cells were incubated for 30 min in the dark. Subsequently, the cells were washed with ice-cold PBS, resuspended in PBS, and analyzed immediately in a flow cytometer (Cytoflex, Beckman Coulter, Indianapolis, IN, USA).

### 2.6. Real-time Polymerase Chain Reaction (RT-PCR)

Vimentin and fibronectin are two important markers for mesenchymal transitions. The expression of vimentin and fibronectin was examined using quantitative real-time PCR (qPCR) for MDA-MB-468, EMT, and MET cells. The cells were collected at different stages of the experimental procedure, such as at the inlet and outlet of the microchannel. For obtaining MET cells, the EMT cells that passed through the channel were collected at the outlet and regrown. The cells were then lysed and total RNA was isolated using an RNA isolation kit. Total RNA (1 µg) was then used to prepare cDNA using a cDNA synthesis kit. qPCR was performed using the primers for vimentin, fibronectin, MDR1 (ABCB1), and GAPDH (Table S1, Supplementary Information). SYBR Green was used as a reporter dye in a Rotor-Gene Q (Qiagen) instrument. The relative expressions of vimentin and fibronectin were calculated by the ΔΔC_t_ method using glyceraldehyde-3-phosphate dehydrogenase (GAPDH) as the endogenous control.

### 2.7. Dual Staining

Trypan blue dye is used to stain membrane-compromised or dead cells, whereas live cells exclude the dye. MDA-MB-468 cells were seeded in a 60 mm cell culture dish in the presence of DMEM and treated with 20 ng/mL of EGF for 24 h. The EMT cells were then washed with PBS, trypsinized, and suspended in DMEM in an Eppendorf tube. Equal volumes of trypan blue dye (10 µL) and the cells were mixed and loaded in the counting chamber. The viable cells (%) were counted using a Countess automated cell counter (Invitrogen). The images highlighting the live and dead cells were also captured using the same instrument. The viability results represented data of triplicate experiments. Acridine orange and ethidium bromide (AO/EtBr) staining was performed to study the viability of the cells passing through the constricted microchannel at four different locations, i.e., at the inlet (1st), while the cells flow through the blockages (2nd), followed by the 7 µm constricted microchannels (3rd), and at the outlet (4th). Briefly, EGF-treated cells were stained with a mixture of AO (100 µg/mL) and EtBr (100 µg/mL) in DMEM for 15 min. After washing with PBS, the cells were trypsinized, mixed with AO/EtBr solution, and passed through the microchannel. When the cells had reached the outlet, the flow pump was stopped and images of the cells at the above-mentioned sites were captured using a Nikon Eclipse Ti-U microscope.

### 2.8. Confocal Imaging to Study the Morphology of Induced EMT Cells

Briefly, 2 × 10^5^ cells were seeded in a 35 mm dish fitted with a coverslip. After attachment, the serum-free medium was added. The cells were treated with 20 ng/mL of EGF for 24 h, then calcein-AM staining was performed. Subsequently, the cells were fixed with 4% formaldehyde and the nuclei were stained with DAPI (4’,6-diamidino-2-phenylindole). Images were obtained in the confocal microscope.

### 2.9. Scratch Assay for Invasion Study

A scratch assay is generally used to observe interactions and migrations among cells. A scratch is marked on a monolayer of cells and subsequent cell migration is captured microscopically at regular time intervals. For this assay, MDA-MB-468 (parental) EMT cells were seeded on 35 mm Petri dishes, whereas EMT cells were treated with 20 ng/mL hEGF. These culture dishes were then scratched with a sterile pipette tip to create a ‘wound’ in the respective dishes. Cell debris was removed by washing with PBS and the plates were kept in an incubator at 37 °C under humidified conditions with 5% CO_2_ for 48 h. The images of the fresh wounds, and also the healing of the wounds, were examined under a Nikon Eclipse Ti-U microscope at 24 and 48 h, respectively, and the corresponding images were examined to differentiate their migration ability.

### 2.10. Cell Viability Assay

To study the viability of the paclitaxel-treated cells, MTT assays were carried out. MDA-MB-468 cells were seeded in a 96-well plate at a density of 5000 cells per well, then the cells were treated with different concentrations of paclitaxel (0–200 nM) for 48 h. After completion of the treatment, the cells were incubated with MTT solution (0.25 µg/mL) in PBS for 2 h. Finally, the MTT solution was aspirated and 150 µL of dimethyl sulfoxide (DMSO) were added to each well. The absorbance of MTT was recorded in a multiplate reader at 570 nm. The cell viability of the treated samples was calculated assuming 100% viability in untreated wells. The results were represented accumulating data from three sets of experiments.

### 2.11. Colony Formation Assay

The colony formation study was performed to investigate whether the EMT cells retained their metastatic ability after passing through the channel. The experiment was designed in such a way that we could compare the number of colonies of the epithelial, EMT, and MET cells along with their paclitaxel-treated counterparts. Epithelial and MET cells were treated with 100 nM paclitaxel for 48 h and a total of 200 cells were seeded for colony formation. In the case of EMT and paclitaxel-treated (EMT + PTX) cells, the cells were treated with EGF or EGF + 100 nM paclitaxel, respectively, for 48 h. After completion of the treatment, the cells were passed through the channel and collected in an Eppendorf tube. The collected cells were counted and dispersed in DMEM in a 12-well plate at a density of 200 cells per well. The plates were incubated for 10 days at 37 °C in an incubator, then the grown colonies of the cells in each well were fixed with 100% methanol and stained with crystal violet. The colonies from each well were counted at 10× magnification under a bright-field microscope (Nikon Eclipse Ti-U, Tokyo, Japan). The results were accumulated based on triplicate experiments.

## 3. Results

### 3.1. Experimental Setup

The basic layout of the microchannel is shown in Figure 2A. The channel was 2.85 mm long between the inlet and outlet sections and its width was 184 μm. At a distance of 700 μm from the inlet, four square blockages of width 30 μm were present. The blockages were unequally spaced along the width of the channel, creating unequal gaps. The gap between the walls of the channel and the first blockage, from either side was 15 μm and the next two blockages were at a distance of 12 μm from the first blockage, leaving a gap of 10 μm between the two center blockages.

At a distance of 700 μm from the blockages, the channel diverged into three parallel channels of width 35 μm and length 300 μm, each of which further reduced to constricted microchannels of width 7 μm and length 150 μm. The channel outlet was at a distance of 1 mm from the constricted channels. Magnified views of the blockage section and the constricted channel section are shown in Figure 2B,C, respectively. The motion of the cells through the blockage section represented their invasiveness through the network of several capillaries. The locomotion of the cells through the entire channel (from inlet to outlet) has been shown in Supplementary video S1.

### 3.2. EGF Induced EMT Transition in MDA-MB-468 Cells

**Figure 3:**
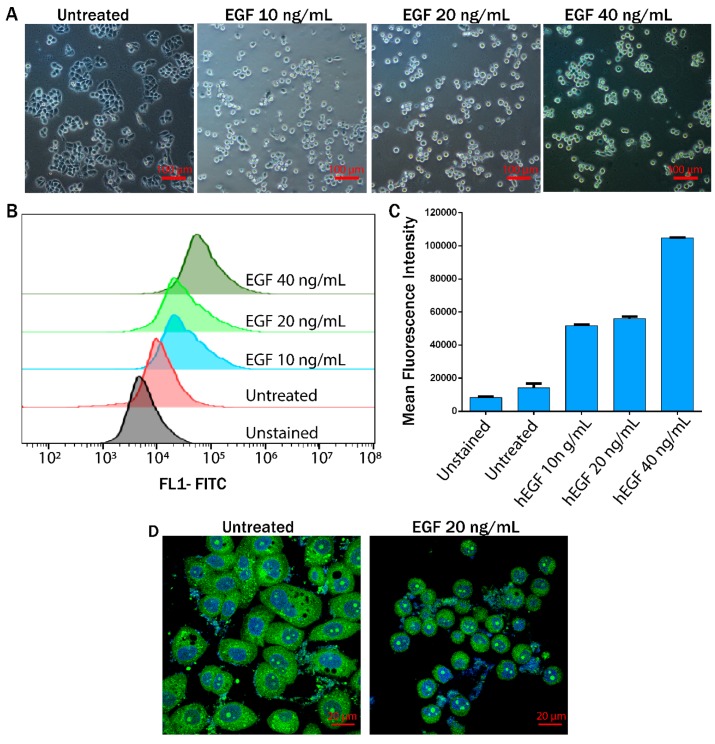
Epithelial to mesenchymal transition (EMT) induction in MDA-MB-468 cells. (**A**) Changes in the morphology of cells treated with increasing concentrations of epidermal growth factor (EGF); (**B**,**C**) histograms from flow cytometry for vimentin expression (**B**), with corresponding mean fluorescence intensity shown in a bar plot (**C**); (**D**) alteration in the cytoplasmic and nuclear morphology studied by calcein-AM DAPI (4’,6-diamidino-2-phenylindole) staining.

EGF induced EMT transition of MDA-MB-468 cells can be monitored by overexpression of the vimentin and N-cadherin that helps in the migration of EMT cells [16,17]. In our experiments, we used EGF to induce EMT to mimic the in vivo conditions. To study the amount of EGF required to convert cells to the mesenchymal state, MDA-MB-468 cells were treated with 10, 20, and 40 ng/mL EGF. After treatment for 24 h, the cells were observed under a microscope for morphological changes. It was observed that untreated cells were in tight contact with each other, but after EGF treatment the cells became rounded losing their contacts (Figure 3A,D). After confirming the morphological changes, a flow cytometric assay was performed to study vimentin expression (for details, see the Experimental section). The cells showed increased expression of vimentin when treated with EGF at 10, 20, and 40 ng/mL (Figure 3B), confirming an EMT. In addition, the viability of these EGF-treated cells was studied using trypan blue staining. From microscopic observations, it was found that cell death was higher in the case of 40 ng/mL EGF treatment.

Therefore, although the maximum vimentin expression was observed at 40 ng/mL EGF, we chose 20 ng/mL of EGF for further experiments. Wound healing assays revealed a greater migratory ability of EMT cells than the untreated cells (referred to as ‘epithelial cells’) (Figure S1).

### 3.3. Flow Dynamics of EMT Cells

**Figure 4:**
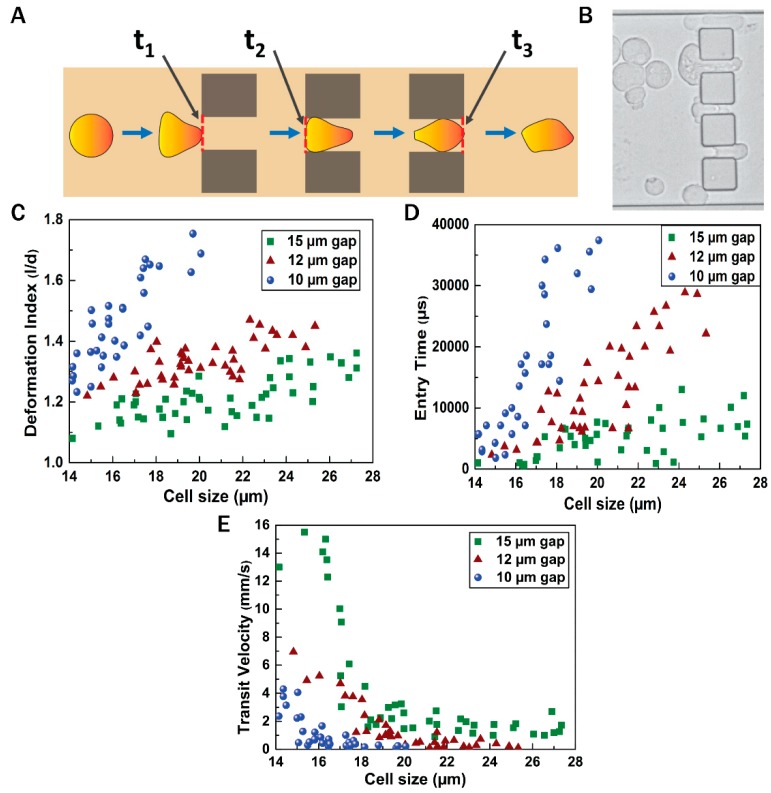
Flow dynamics of cells through blockages. (**A**) Stepwise motion of the cell through a blockage; (**B**) microscopic view of invasion of cells through the gaps between blockages (**C**–**E**) deformation index, entry time, and transit velocity of the cells through the gaps of varying sizes between blockages, respectively.

Figure 4 shows the flow dynamics of the EMT cells when they pass through the gaps of various sizes between the 30 μm blockages. Figure 4A illustrates the various time instants recorded during the experiments while the cells passed through any gap. Time *t*_1_ was taken at the instant when the cell was just about to enter the gap (the cell front touches the entry line). This was followed by time *t*_2_, which is the instant when the entire cell had entered the gap (the rear of the cell touched the entry line). Finally, we recorded time *t*_3_ when the cell was about to exit from the gap (the front of the cell touched the exit line). Based on the values of *t*_1_, *t*_2_, and *t*_3_, the deformation index, entry time, and transit velocity of the cell, while moving through the gaps, were calculated.

A microscopic image of the cells passing through the gaps between the blockages is shown in Figure 4B. Figure 4C shows the deformation index of the cells while passing through the gaps. The cell sizes varied in the range of 14–28 μm diameter and the gap sizes varied as 10, 12, and 15 μm. Figure 4D shows the entry time required for the cells of different sizes to enter the gap, and the velocity with which the cells transit through the gaps has been shown in Figure 4E. It was observed that through the 10 μm gap, comparatively smaller cells (size less than ~20 μm) tended to pass through and, owing to increased confinement, the deformation index and entry time of the cells passing through the 10 μm gap were very high compared with the 12 and 15 μm gaps. The gaps of 12 and 15 μm allowed more cells to pass through. For any particular cell size, the deformation index and entry time were minimum, and the transit velocity was maximum in the 15 μm gap. Supplementary video S2 depicts the motion of the EMT cells through the gaps between the blockages in the channel. It was observed that a cell of diameter 20 μm (approx) exhibited deformation index of 1.69, 1.31, and 1.21 with the corresponding entry time of 37,428, 14,334, and 7667 μs, and possessed transit velocity of 0.227, 0.326, and 1.47 mm/s, while traversing through the 10, 12, and 15 μm gap, respectively.

**Figure 5:**
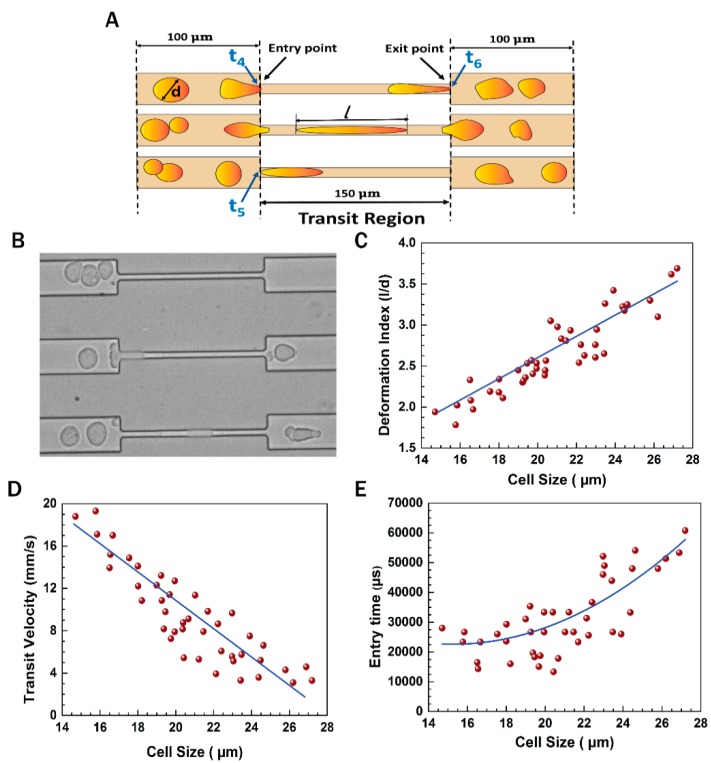
Flow dynamics of cells through a constricted 7 μm channel. (**A**) Stepwise motion of the cells through the constricted channel; (**B**) microscopic image of cells passing through constricted microchannel; (**C**–**E**) deformation index, entry time, and transit velocity of the cells through the 7 μm constricted passage, respectively.

As mentioned earlier (Figure 2), each of the three constricted passages were of equal length (150 μm) and width (7 μm). Figure 5 shows the flow dynamics of the cells through the constricted 7 μm microchannels. Time instants *t*_4_, *t*_5_, and *t*_6_ were observed when a cell passed through any of the constricted paths. As shown in Figure 5A, *t*_4_ is the time instant when the cell front touched the entry point in any channel, *t*_5_ is the time instant when the entire cell has just entered the constricted passage and its rear touched the entry point, and *t*_6_ is the time instant when the deformed cell’s front touched the exit point. The entry time was calculated by subtracting *t_4_* from *t*_5_. The ratio of the maximum elongation length (*l*) to the undeformed cell diameter (*d*) was calculated as the deformation index. The average transit velocity was obtained by dividing the distance travelled (150 μm) by the time taken (*t*_6_–*t*_4_). A microscopic view of the cells flowing through the constricted channels is shown in Figure 5B. Figure 5C shows the deformation index of the cells through the constricted 150 μm long passage. The cell sizes varied in the range of 14–28 μm. It was observed that the large cells underwent enhanced elongation compared with small cells. The transit velocity and entry time of the cells are shown in Figure 5D,E, respectively. It is noted that large cells took more time to accommodate themselves inside the constricted passage, exhibiting an enhanced entry time and a lower transit velocity.

A typical cell of size 19 μm diameter showed a deformation index of 2.45, transit velocity of 12.3 mm/s, and entry time of 31,000 μs, while moving through one of the constricted sections of the channel. The blue lines in the plots depict the general trend of the nature of the cells. These are the best fitted curves obtained from the data points in the graph. Supplementary video S3 depicts the motion of the cancer cells through the constricted microchannels.

### 3.4. Epithelial to Mesenchymal and Mesenchymal to Epithelial Transitions

Epithelial cells possess tight contacts with neighboring cells, and thus express proteins required for adherence (E-cadherin, occludin), whereas EMT-transformed cells become loosely attached, gaining migratory properties. In our experiments, we used vimentin as a standard EMT marker to confirm the epithelial or mesenchymal status of the cells [18]. The presence of EMT in MDA-MB-468 cells, and also the viability of the cells at the outlet, can be used to study the behavior of these cells in blood vessels.

**Figure 6:**
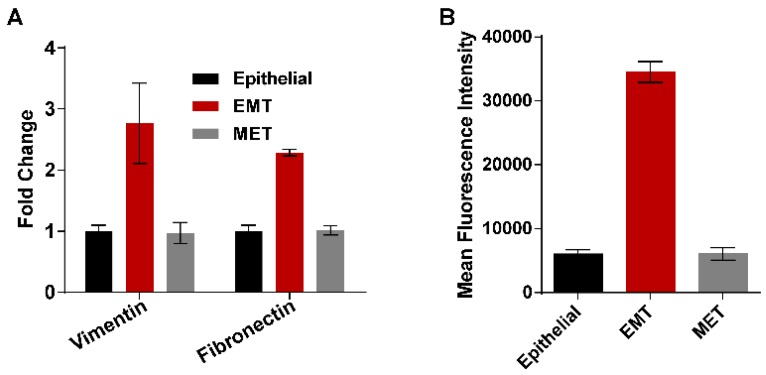
Gene expression studies by real-time PCR and flow cytometry. (**A**) Comparison of expression of vimentin and fibronectin genes in epithelial, EMT, and mesenchymal to epithelial transition (MET) cells; (**B**) vimentin expression studied at the protein level using anti-vimentin antibody in flow cytometry.

EMT was induced in presence of EGF. However, in the absence of EGF during movement, downregulation of vimentin and fibronectin were observed in the cells collected at the outlet, which defines possible reverse transition to MET. Therefore, EMT-induced cells were collected at the outlet of the microchannel (referred to as MET cells) and studied for possible MET characteristics. From gene expression studies (Figure 6), it was confirmed that EGF-treated cells showed a 2.7–fold higher expression of vimentin protein compared with untreated epithelial cells, confirming the epithelial to mesenchymal transition of MDA-MB-468 cells. Similarly, fibronectin expression also increased two-fold (Figure 6A) [12]. These events are similar to those that occur at the primary site of the tumor, where the expression of the epithelial marker decreases and mesenchymal marker protein expression increases simultaneously during EMT [19]. We isolated these mesenchymal cells and passed them through the microchannel, which mimicked the entry and movement of the mesenchymal cells in blood vessels. In general, when the mesenchymal cells reach the bloodstream, they travel to different parts of the body and start to be converted into the epithelial state. Hence, in our experiments, cells passing through the microchannel were collected at the outlet and were grown to study their MET characteristics. Surprisingly, vimentin and fibronectin expression reached its basal level as in epithelial cells, confirming the complete mesenchymal to epithelial transition.

### 3.5. Viability of EMT Cells

**Figure 7:**
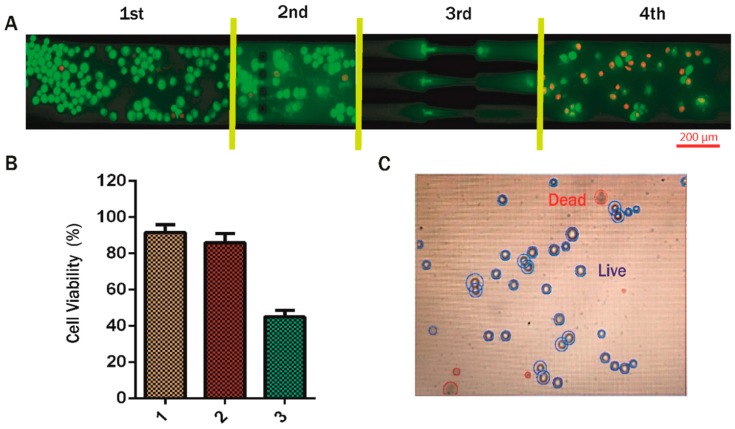
Cell viability assessment. (**A**) Acridine orange and ethidium bromide (AO/EtBr)-stained images at different sections of the channel; (**B**) comparison of the percentage of EMT cells viable after passing through the constriction with the initial conditions and with cells maintained in the same environment for the same period of time but not passed through the constriction. Cell condition 1 refers to the initial EMT cells, cell condition 2 refers to mock experimental control EMT cells kept in the same environment without passing through the channel, and cell condition 3 refers to the cells collected from the outlet of the microchannel; (**C**) image of stained cells obtained from the Countess automated cell counter.

As mentioned previously, the microchannel consisted of several obstructions, and the cells passing through the channel underwent deformation and pronounced morphological changes, decreasing the percentage of viable cells at the outlet [20]. Mesenchymal transformed MDA-MB-468 cells were collected by trypsinization before starting the experiment. The fluorescent images in Figure 3D confirmed the membrane integrity of EMT cells. For studying the viability, we performed AO/EtBr staining. Figure 7A shows the qualitative images of the cells at different sections of the channel. At the inlet, all of the cells were evenly stained with AO, with a few red dots indicating EtBr fluorescence due to dead cells. On passing the gaps of 10, 12, and 15 µm (between blockages), the cells were observed to be mostly alive. However, once the cells had passed through the 7 µm constriction and reached the outlet, almost 50% of the cells took up EtBr, confirming cell death. Similar events resulting in cell apoptosis have been observed during migration and invasion through blood capillaries smaller than their own diameter [21,22]. Quantitative estimation of the cell death during the flow was estimated by trypan blue staining, which further confirmed the presence of live cells at the outlet of the channel. Figure 7B indicates that around 50% of the cells were alive at the outlet.

### 3.6. Clonogenicity of EMT Cells

**Figure 8:**
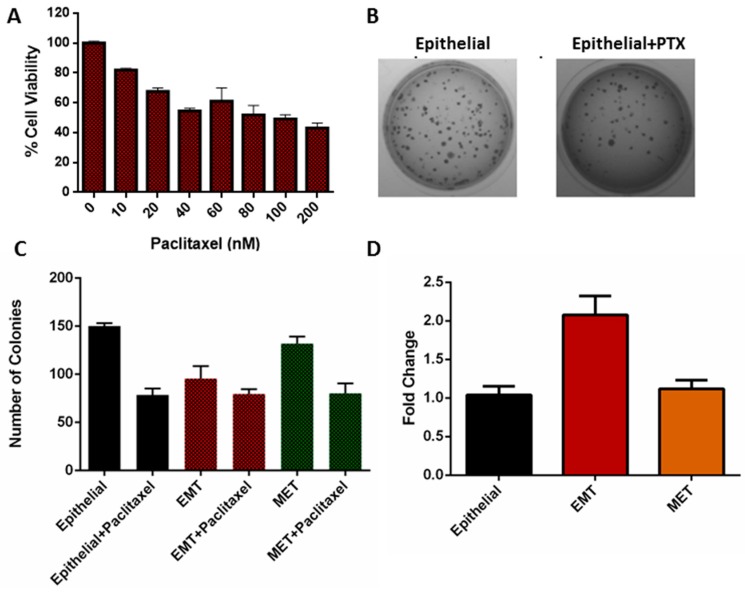
(**A**) MTT (3-(4,5-dimethylthiazol-2-yl)-2,5-diphenyltetrazolium bromide) assay results of the paclitaxel treatment of MDA-MB-468 cells for 48 h; (**B**) representative photograph of the colony formation assay of epithelial and drug-treated epithelial cells; (**C**) comparison of the number of colonies formed by the paclitaxel-treated epithelial, EMT, and MET cells with their respective controls; (**D**) real-time PCR assay results showing MDR-1 expression in epithelial, EMT, and MET cells.

The ability of a single cell to grow and form colonies was studied by clonogenic or colony formation assay. Initially, MDA-MB-468 (epithelial) cells were treated with different concentrations of paclitaxel (PTX) to study the antiproliferative properties of the PTX. From the MTT assay results for 48 h treatment (Figure 8A), it was observed that PTX was able to inhibit the proliferation of the cells in a dose-dependent manner and the IC_50_ of the PTX was found to be 98 ± 4 nM. Next, epithelial, EMT, and MET cells were treated with PTX and an equal number of cells were seeded to form colonies. All the cells treated with EGF, i.e., EMT and EMT treated with PTX, were passed through the channel and seeded for colony formation. Epithelial and MET cells, and also their drug-treated counterparts, were not passed through the microchannel. It was observed from Figure 8B,C that a total of 150 ± 3 colonies were formed from untreated epithelial cells, whereas epithelial cells treated with PTX showed 78 ± 5 colonies, confirming the antiproliferative effect of the PTX. Surprisingly, the number of colonies formed in EMT cells induced by EGF was 95 ± 10, whereas EMT cells treated with PTX formed 79 ± 4 colonies. Similarly, untreated MET cells formed 131 ± 6 colonies, whereas MET cells treated with PTX developed 80 ± 8 colonies. From the results, it was concluded that both epithelial and MET cells treated with PTX showed a decreased number of colonies due to the effect of the drug. In contrast, EMT cells treated with PTX did not show a significant reduction in colony formation. Early reports suggested that EMT cells become drug resistant by acquiring increased drug efflux pumps, leading to a smaller amount of the drug being available for therapeutic action inside the cells [23]. In this regard, we analyzed the expression of the MDR1 in the epithelial, EMT, and EMT cells. MDR1 protein forms drug efflux pumps in the cell membrane to avoid cell death from therapeutic drugs. The results of the real-time PCR analysis in Figure 8D show that the EMT cells possess 2-fold higher expression of the MDR1 as compared to epithelial cells. The expression remains similar for MET cells. Hence, the increased number of colonies in EMT cells after PTX treatment may be attributed to the acquired resistance of the EMT cells due to increased MDR1 expression. To the best of our knowledge, this is the first report in which the hydrodynamic behavior of EMT cells is correlated with drug-resistant metastatic phenomena of breast cancer cells.

## 4. Discussion

The motion of the cells through the blockage section of the microchannel represents invasive behavior of the cells through the basement membrane to enter the bloodstream. On the other hand, the motion of the cells through the constricted channels represents their motion through the network of blood capillaries. Our experimental design of microchannels and working protocols showed stepwise movements of EMT cells similar to that of in vivo metastatic cells (Figure 5). Starting from standardization of EGF dose (20 ng/mL), induction of EMT and susbsequent gene expression profiling of vimentin and fibronectin were perfomed systematically (Figure 3 and Figure 6) before conducting the flow experiments. The scratch assays (Figure S1) supported the migratory ability of EGF induced EMT cells, as evident from other reports [24]. It is to be mentioned here that during the initial stages of EMT, biochemical changes inside the cells cause alterations in the morphology of the cells, which was observed in Figure 3 [25]. Normally, epithelial cells are tightly attached to the basement membrane; however, during EMT, cells lose their contact with the attached surface and undergo migration and invasion. Both the migratory and invasive properties are associated with the expression of the genes from the signaling pathways involved. Certain epithelial marker proteins such as E-cadherin, claudin, and occludin are downregulated in the mesenchymal state, whereas mesenchymal markers (N-cadherin, vimentin, Snail) are upregulated [26]. Such alterations in vimentin and fibronectin expression confirmed EMT formation (Figure 6). Interestingly, these EMT cells passing through the gaps of various blockages and the constricted capillaries revealed some important hydrodynamic parameters, like deformaton index and transit velocities, as depicted in Figure 4 and Figure 5. Such parameters are important to understand the ability of metastatic cells to move through several hindrances while reaching the distant secondary site from the primary origin. It is quite intriguing that the cells undergo deformation several times during such movement through microcapillaries. In reality, the cancer cells pass through several such hindrances (as demonstrated in Figure 4B and Figure 5B) while moving from a primary site to reach a secondary site during metastasis. Such cancer cells get deformed on multiple occasions during their movement through a system of capillaries. Importantly, having been deformed some cells still remain viable for spreading cancer. Our experimental design has explained deformability and survival behavior of EMT while passing through several barriers and constricted portion of microchannel.

A significant population of EMT cells retained viability to spread cancer, as evident from the trypan blue staining experiment (Figure 7). When the disseminated mesenchymal cells reach a secondary site (site of metastases) by traversing the network of blood capillaries, they may undergo a reverse transition (MET) as metastases largely recapitulate an epithelial-like pathology similar to the corresponding primary tumor [27]. It is very important to understand that the environment faced by the cells at the secondary site completely differs from the cells at primary site. In our microchannel, we considered the outlet as a secondary site. This experimental design led us to analyze the morphology and properties of the cells collected from the outlet. The real-time PCR and flow cytometry results for vimentin and fibronectin expression described in Section 3.4 (Figure 6) have shown that the cells collected from outlet completely returned back to the epithelial state after incubation in a growth factor-free medium. As such, there was no evidence that some of the cells are residing at mesenchymal state. Similarly, the MDR-1 expression of the MET cells also reached the basal levels, indicating the complete mesenchymal to epithelial transition. The MTT assay provided the required drug concentration for treating cells in various transition phages. The IC_50_ value of PTX treated MDA-MB-468 cells was obtained by this assay. The same dose of PTX was further used to treat EMT and MET cells (cells collected at the outlet). Finally, EMT alone, and EMT and MET treated with PTX separately, were compared for their colony formation abilities. Virulence of EMT with increased number of colonies and resistance to PTX treatment with high MDR1 expression (Figure 8) deciphered drug-resistant behavior of metastatic EMT cells passing through microchannel. The strength of our study is not only limited to movement of EMT cells mimicking in vivo conditions, but it also deciphers the drug resistant properties of EMT, as well as reverse transition phenotype, in terms of gene expressions.

## 5. Conclusions

This paper describes the flow of EMT cells in constricted microcapillaries while retaining their metastatic potential. The EMT induced MDA-MB-468 cells regained their epithelial nature with potential ability to grow and divide after passing through the microchannel traversing through several barriers. During such migration, the cells undergo deformation and transitions that were established experimentally. Higher expression of EMT markers such as vimentin and fibronectin and enhanced migratory ability of the cells, confirmed by the scratch assay, described their metastatic behaviors. While exhibiting metastasis, cells invade through surrounding tissue layers to enter the blood circulation. These aspects of the cell migration were studied in microchannels possessing barriers in the form of blockages and constricted passages to mimic in vivo conditions. In experimental setup, hydrodynamic properties of the moving EMT cells were evaluated, which revealed pronounced deformability of the cells. Surprisingly, the viable cells collected at the outlet were transformed into MET with the ability to form colonies, similar to a condition for formation of secondary tumor in metastasis. Further, the effect of a chemotherapeutic drug (paclitaxel) on the cells revealed higher expression of the multidrug resistance MDR-1 gene in EMT cells, which possibly enhanced their drug resistance, than epithelial and MET cells. Our experimental findings provide insight into flow dynamics of EMT cells and their drug resistant behaviors during the progress of metastasis. The current information, resembling migration properties of metastatic cells through tissues and blood vessels in vivo, would be beneficial to devise therapeutic strategies in future.

## Supplementary Materials

The following are available online at https://www.mdpi.com/2077-0383/8/8/1194/s1. Table S1: Primer sequences used for real-time PCR study. Figure S1: Wound healing assay results revealing migration of the EGF treated cells. Supplementary video S1 shows the flow of cells in the entire channel from the inlet to the outlet reservoir. Supplementary video S2 shows the motion of cells through the gaps between the blockages in the channel. Supplementary video S3 shows the motion of cells through the constricted passages in the channel.
