# Supplementary material for: Deciphering Hydrodynamic and Drug-Resistant Behaviors of Metastatic EMT Breast Cancer Cells Moving in a Constricted Microcapillary
Source: J Clin Med. 2019 Aug 9;8(8):1194. doi: 10.3390/jcm8081194 (PMC6722803; doi:10.3390/jcm8081194)
Supplement: Supplementary file 1 [file jcm-08-01194-s001.zip › jcm-555599-supplementary/Supplementary Informaton.docx]

**Supplementary**

**Table 1.** Primer sequences of the genes used for real-time PCR analysis.

| **Gene** | **Forward (5’to 3’)** | **Reverse (5’to 3’)** |
| --- | --- | --- |
| Vimentin | AGTCCACTGAGTACCGGAGAC | CATTTCACGCATCTGGCGTTC |
| Fibronectin | GGTGACACTTATGAGCGTCCTAAA | AACATGTAACCACCAGTCTCATGTG |
| GAPDH | GAAATCCCATCACCATCTTCCAGG | GAGCCCCAGCCTTCTCCATG |
| MDR1 | GGGAGCTTAACACCCGACTTA | GCCAAAATCACAAGGGTTAGCTT |


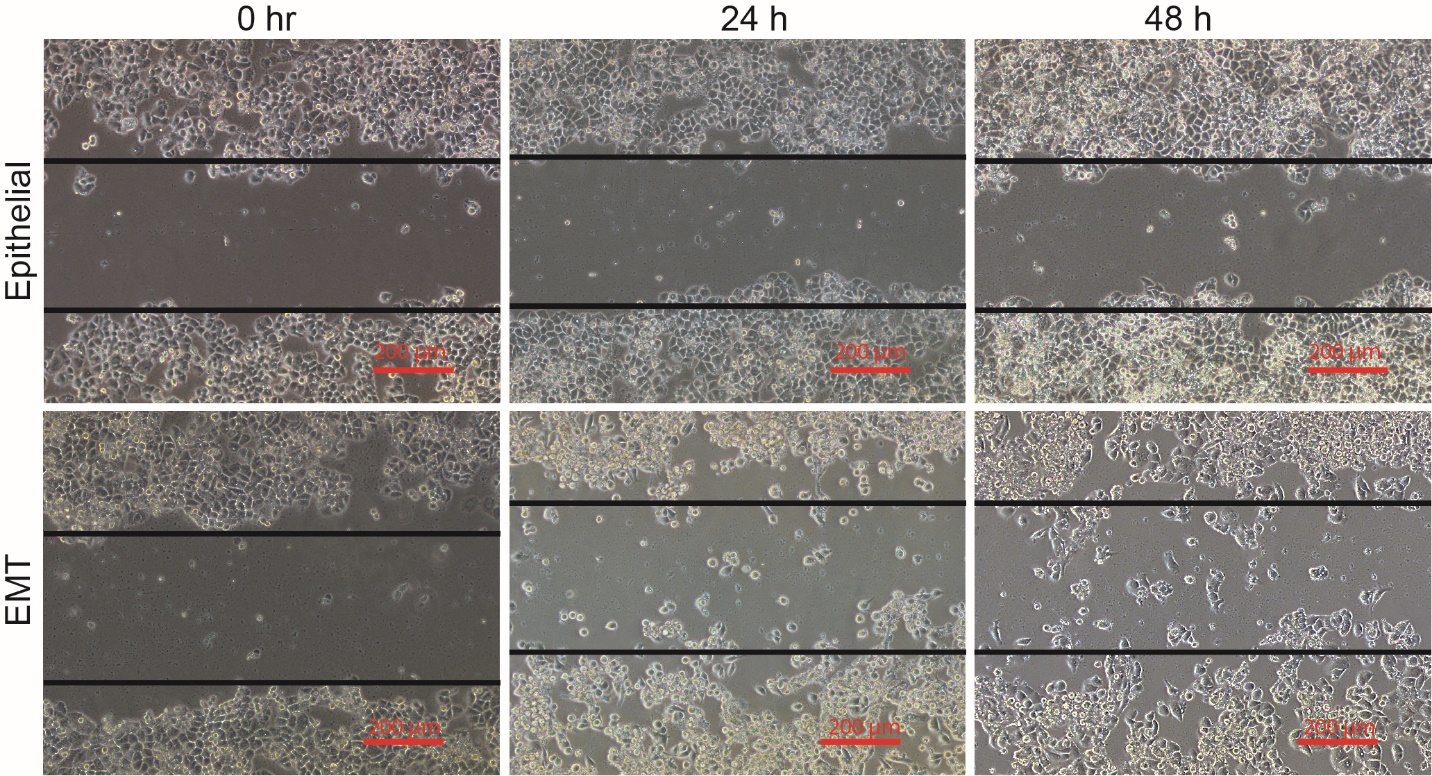


**Figure 1.** Wound healing assay results revealing migration of the EGF treated cells.

Supplementary video S1. shows the flow of cells in the entire channel from the inlet to the outlet reservoir.

Supplementary video S2. shows the motion of cells through the gaps between the blockages in the channel.

Supplementary video S3. shows the motion of cells through the constricted passages in the channel.
